# Manipulation of *StPTST1* Affects Starch Content and Physicochemical Properties of Potato (*Solanum tuberosum* L.)

**DOI:** 10.3390/plants14213351

**Published:** 2025-10-31

**Authors:** Zhenming Shi, Xiaoyi Cao, Hongyuan Zhou, Ting Yu, Yi Shang, Jianfei Xu, Dongli Gao

**Affiliations:** 1 Key Laboratory for Potato Biology of Yunnan Province, College of Life Sciences, Yunnan Normal University, Kunming 650500, China; shimingoo@126.com (Z.S.); 15126636182@163.com (X.C.); zhouhongyuang@163.com (H.Z.); 15751998743@163.com (T.Y.); shangyi@caas.cn (Y.S.); 2 College of Life Sciences, Southwest United Graduate School, Kunming 650092, China; 3 State Key Laboratory of Vegetable Biobreeding, Institute of Vegetables and Flowers, Chinese Academy of Agricultural Sciences, Beijing 100081, China

**Keywords:** potato, *StPTST1*, resistant starch, physicochemical properties, nutritional quality

## Abstract

Foods rich in amylose and resistant starch (RS) hold great potential for improving human health. Granule-bound starch synthase (GBSS) is a key enzyme for amylose biosynthesis and its interaction partner, PROTEIN TARGETING TO STARCH1 (PTST1), has been characterized. In this study, we generated overexpression and knockout transgenic plants of *StPTST1* to investigate its effect on starch content and physicochemical properties. Aligning with the presence of carbohydrate-binding module in the protein, *StPTST1* possesses starch-binding capacity. *stptst1* knockout mutants showed a reduction in both total starch and amylose contents in tubers. Analysis of the pasting properties showed that peak viscosity (PV), trough viscosity (TV), breakdown viscosity (BV), final viscosity (FV), and setback viscosity (SV) were all increased in the mutants compared to that in the WT plants. Overexpression of *StPTST1* led to an increase in the contents of amylose, RS, and total starch. Moreover, the proportion of short chains (0 < DP < 32) in amylopectin of *StPTST1*-overexpressing plants was reduced. These data demonstrated that both *stptst1* mutants and *StPTST1*-overexpressing plants were altered in starch content and physicochemical properties. Elucidating the function of StPTST1 deepens our understanding of starch biosynthesis in potato and highlights its potential for enhancing potato nutritional quality.

## 1. Introduction

Starch, a plant-derived carbohydrate, serves as a crucial energy source for humans and livestock. Potato starch, extracted from the tubers of *Solanum tuberosum* L. (a member of the Solanaceae family), is one of the most widely used starches in both food and non-food industries. It possesses unique physicochemical properties, such as high transparency, low gelatinization temperature, and excellent freeze–thaw stability, which distinguish it from other commercial starches like corn starch or wheat starch [1]. It acts as a thickener, stabilizer, and gelling agent in various food products, including soups, sauces, and gluten-free baked goods [2].

Resistant starch (RS) refers to the type of starch that cannot be absorbed or digested in the small intestine of healthy humans [3]. Nevertheless, intake of RS exerts highly positive effects on human health; it can improve carbohydrate and lipid metabolism disorders [4], and undergoes fermentation in the intestine to produce large quantities of short-chain fatty acids that are beneficial to the human body [5]. Therefore, increasing the RS content in potato is of great significance for enhancing the health of populations relying on potato as a staple food. The RS content is mainly determined by the intrinsic properties of starch. In corn starch, high-amylose varieties have a significantly higher proportion of RS compared to low-amylose varieties [6]. The ratio of amylose to amylopectin significantly affects the formation of RS; generally, a higher ratio is associated with an increase in RS content [7]. Although potato starch has a lower amylose content than rice starch, it can generate a higher amount of RS, making it more suitable for RS production [8].

Starch synthesis is a complex enzymatic process. Starch synthases are categorized into Granule-bound starch synthase (GBSS) and soluble starch synthases (SSS) based on their functions and characteristics [9]. GBSS is responsible for amylose biosynthesis, while SSS promotes the elongation of amylopectin chains [10]. Reducing GBSS activity in potato impairs amylose synthesis [11]. It has been reported that knockout or loss-of-function *GBSS* mutants synthesize waxy starch (amylose-free starch); for instance, hexaploidy wheat produces such starch, and potato mutants with this trait develop tubers without amylose [12,13]. In rice, there are two isoforms of GBSS, namely GBSSI and GBSSII. GBSSI is mainly involved in amylose synthesis in endosperm, while GBSSII plays a role in amylose synthesis in leaves [14]. Unlike rice, only GBSSI exists in potato [15].

Modulating the starch biosynthetic pathway represents an effective strategy for breeding high-amylose crops, for instance, by enhancing *GBSS* expression and inhibiting or knocking out genes encoding sucrose synthase, starch branching enzyme, debranching enzyme, and glucan-water dikinase [16]. In *Arabidopsis thaliana*, a non-catalytic carbohydrate-binding protein PROTEIN TARGETING TO STARCH1 (PTST1) was identified. It acts as a key factor in localizing GBSS to starch granules and regulates amylose biosynthesis in leaves [17]. Barley PTST1 can interact with the GBSSIa protein [18]. A homologous protein, GBSS-binding protein (OsGBP), has been found in rice, which interacts with both GBSSI and GBSSII and is co-localized on the surface of starch granules [19]. Additionally, in rice, the FLOURY ENDOSPERM6 (FLO6) protein is a homolog of AtPTST2. OsFLO6 directly interacts with isoamylase 1 in rice seeds, thereby affecting starch synthesis and starch granule formation [20]. We have proved that GBSSI interacts with StPTST1 [21]. The main aims of this study were to evaluate the effects of *StPTST1* knockout and overexpression on starch content and physicochemical properties.

## 2. Results

### 2.1. StPTST1 Binding to Starch

**Figure 1:**
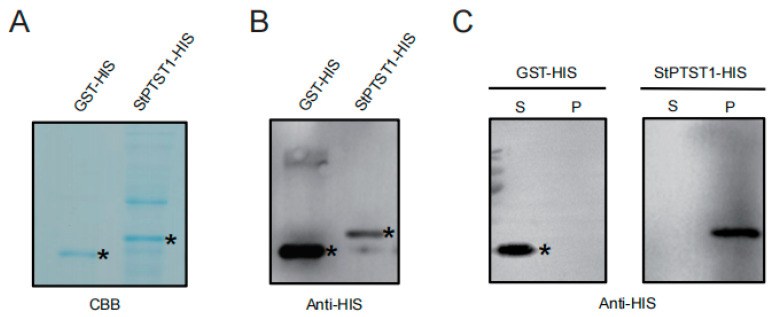
PROTEIN TARGETING TO STARCH1 (StPTST1) binding to starch. (**A**) The HIS fusion proteins of the GST and StPTST1 were run on sodium dodecyl sulfate-polyacrylamide gel electrophoresis (SDS-PAGE), and stained with coomassie brilliant blue (CBB). (**B**) HIS fusion proteins were probed with an anti-HIS antibody to identify the bands corresponding to fusion proteins. (**C**) HIS fusion of StPTST1 was examined for their ability to co-sediment with total starch. The supernatant (S) and pellet fractions (P) were blotted to a polyvinylidene fluoride (PVDF) membrane and probed with an anti-HIS antibody. Asterisks indicate the target bands in the lanes corresponding to the GST-HIS and StPTST1-HIS recombinant proteins.

Carbohydrate-binding module 48 (CBM48) is typically known to bind carbohydrates such as starch and other polysaccharides. StPTST1 contains a CBM48 domain at the 170-252 aa. We performed an in vitro starch-binding assay using the fusion recombinant proteins StPTST1-HIS and GST-HIS, with GST-HIS serving as the negative control (Figure 1A,B). The results showed that the StPTST1-HIS protein was able to bind to starch, while the GST-HIS protein was mainly retained in the supernatant fraction (Figure 1C). The results confirmed that StPTST1 bound to starch.

### 2.2. Knockout of StPTST1 Affected Starch Content and Starch Physicochemical Properties

Transgenic seedlings of CIP065 regenerated via callus induction frequently undergo chromosome doubling. Ploidy detection was performed on the target-edited positive plants, yielding two diploid plants designated as *stptst1*-214 and *stptst1*-267 (Figure S1). To detect the editing efficiency of the target sites in the mutant plants, the target sites were amplified and sequenced. The results showed that for *stptst1*-214, all the 10 single clones were edited, and the mutation included a 4 bp deletion at target 1 and a 1 bp insertion at target 2. The mutations disrupted the coiled-coil structure, but the CBM48 domain remained unchanged (Figure S2A,C). *stptst1*-267 shows six monoclonal instances of edition with two mutation types: one involving a 1 bp insertion at target 2 and another consisting of a 3 bp deletion from target 1 along with the aforementioned insertion at target 2. The mutations resulted in premature termination of protein expression (Figure S2B,C).

**Figure 2:**
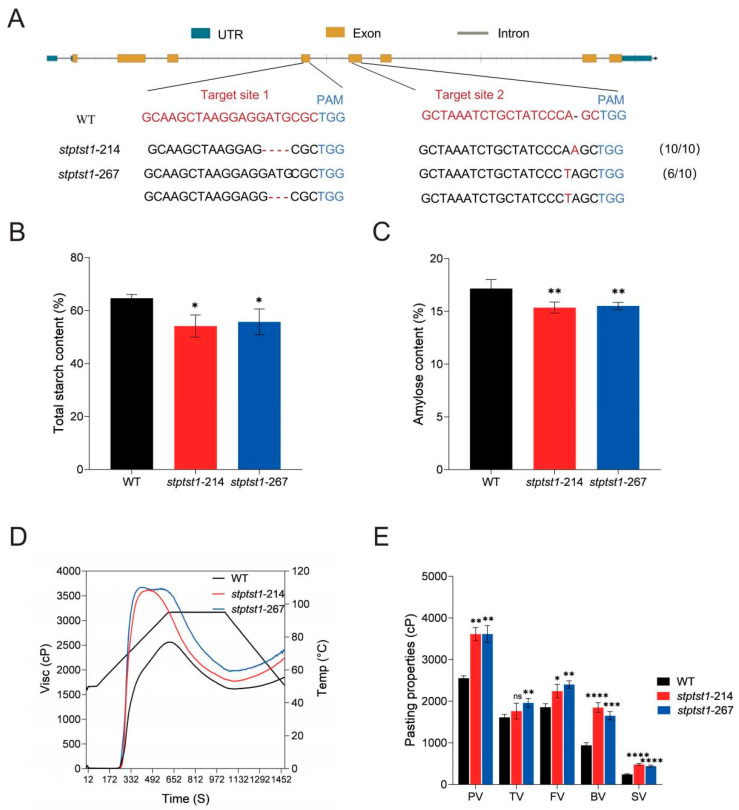
Analysis of the starch content and starch physicochemical properties of the *stptst1* mutants. (**A**) The mutation types of *stptst1*-214 and *stptst1*-267 were identified by direct sequencing. Nucleotide insertions in mutant alleles are indicated by a red mark, and deletions are indicated as a gap. The CRISPR/Cas9 PAM motifs are indicated by blue letters. (**B**) Total starch content. (**C**) Amylose content. (**D**) Pasting properties of tuber starch determined by rapid visco analyzer (RVA). The black line indicates the temperature fluctuation during the measurements. The viscosity value at each temperature is the average of three replicates. (**E**) Pasting profiles of tuber starch. cP, centipoise. Values are means ± SD from three biological replicates. Asterisks indicate statistical significance between WT and mutants. “ns” indicates no significant difference, as determined by ANOVA (* *p* < 0.05, ** *p* < 0.01, *** *p* < 0.001, **** *p* < 0.0001, n ≥ 3).

The *stptst1*-214 and *stptst1*-267 mutants did not exhibit significant differences in either tuber size or number compared to the wild type (Figure S3). The percentages of total starch and amylose in tubers were significantly decreased in the *stptst1*-214 and *stptst1*-267 mutants compared to the wild type (Figure 2B,C). The *stptst1*-214 mutant leaves were stained to visually observe the changes in starch content. When the proportion of amylose in starch is high, the starch exhibits a blue-black color. When the proportion of amylopectin is high, the starch shows a reddish-brown color. Compared to the WT leaves, the *stptst1*-214 mutant leaves exhibited a reddish-brown coloration, indicating lower amylose accumulation. Observation of the stained leaves found that chloroplasts in the mesophyll cells of wild-type plants contained one or more large starch granules in their central region, whereas chloroplasts in *stptst1*-214 mutant plants contained smaller starch granules, and some chloroplasts lacked starch granules entirely (Figure S4). Analysis of the viscosity of starch showed that mutants took a shorter time to reach peak viscosity compared to WT, with *stptst1*-214 and *stptst1*-267 at 7.40 min and 6.54 min, respectively, while WT reached it at 10.30 min (Figure 2D). Notably, the starch in mutants exhibited a higher PV, TV, FV, BV, and SV than those in WT (Figure 2E). Thus, the pasting properties of tuber starch were changed in the mutants.

### 2.3. Overexpression of StPTST1 Led to Enhanced RS Content and Reduced Proportion of Short Chains

**Figure 3:**
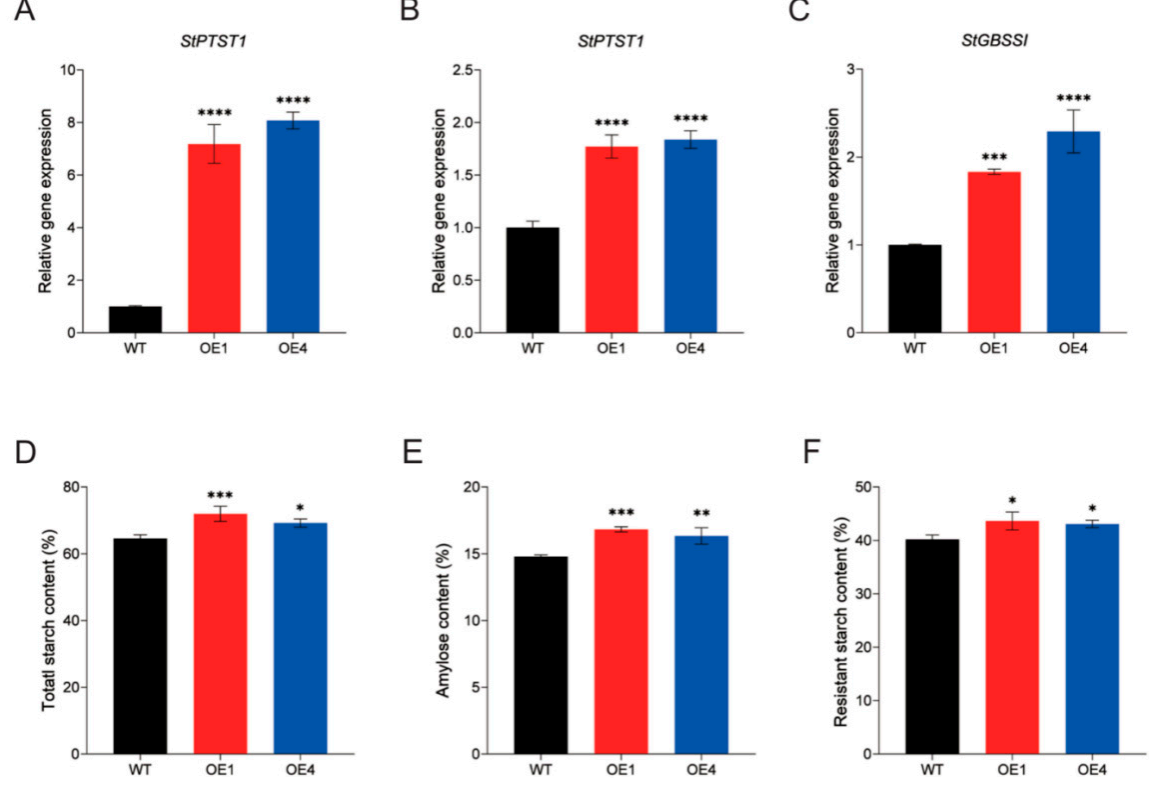
Evaluation of gene expression and starch physicochemical properties of *StPTST1* overexpression plants. (**A**) Relative gene expression of *StPTST1* in leaves from WT and overexpression plants, and (**B**,**C**) *StPTST1* and Granule-bound starch synthase I (*GBSSI*) gene expression in tubers. (**D**) Total starch content, (**E**) amylose content, and (**F**) resistant starch content. Error bars represent the SDs of biological replicates (n = 3). Asterisks indicate the statistical significance between overexpression plants and WT determined by ANOVA (* *p* < 0.05, ** *p* < 0.01, *** *p* < 0.001, **** *p* <0.0001).

Two *StPTST1* overexpression lines were generated, with 6-8-fold higher expression in their leaves compared with that of WT (Figure 3A). Examination of the mRNA levels of *GBSSI* and *StPTST1* in tubers revealed that both genes were upregulated in overexpression lines (Figure 3B,C). The contents of total starch, amylose, and RS in the tubers of transgenic plants and WT were measured. The total starch contents in OE1 and OE4 were significantly higher than that in WT, with an increase of 7.38% and 4.62% for OE1 and OE4, respectively (Figure 3D). The amylose contents showed a similar trend, with an increase of 2.04% for OE1 and 1.55% for OE4 (Figure 3E). Measurement of the RS content showed enhanced accumulation in overexpression lines; OE1 and OE4 had a respective increase of 3.45% and 2.89% than WT (Figure 3F). These results are consistent with previous studies showing that amylose content had a strong positive correlation with RS [22,23,24].

**Figure 4:**
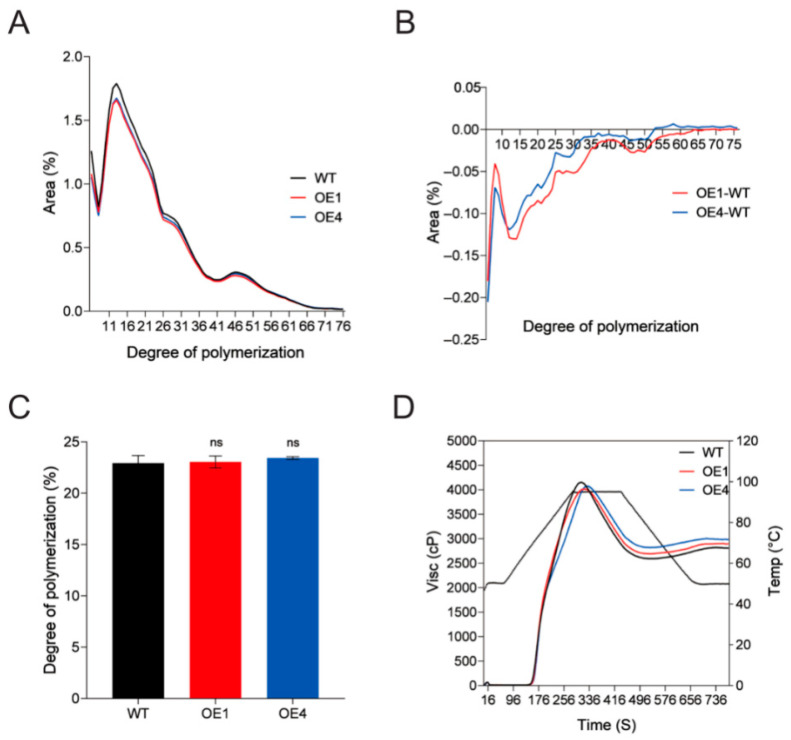
Chain length distribution (CLD) analysis of amylopectin and starch pasting properties in *StPTST1* overexpression plants. (**A**) CLD of the amylopectin after normalization to the total peak area. (**B**) Difference in CLD between overexpression plants and WT. Three biological replicates were performed. (**C**) Average degree of polymerization. (**D**) Rapid Visco Analyser (RVA) pasting profiles of starch. Error bars represent the SDs of biological replicates (n = 3). “ns” indicates no significant difference (ANOVA, *p* > 0.05).

The normalized chain length distribution (CLD) profiles of overexpression and WT lines are presented in Figure 4A. Compared with WT, the overexpression lines showed a marked reduction in the proportion of short chains with degree of polymerization (DP) in the range of (0 < DP < 32), along with no significant changes in medium chains (33 < DP < 60) and long chains (DP > 60) (Figure 4B). The average chain length of amylopectin chains in overexpression lines was not significantly different from that in WT (Figure 4C). The viscosity characteristics of starch from potato tubers of overexpression lines and WT were determined. Rapid Visco Analyser (RVA) curves revealed that the overexpression lines and WT lines showed similar patterns (Figure 4D), and the physicochemical parameters including peak viscosity (PV), trough viscosity (TV), breakdown viscosity (BV), final viscosity (FV), and setback viscosity (SV) were not considerably different between WT and the mutants (Figure S5).

## 3. Discussion

Potatoes are widely consumed vegetables globally and serve as a staple food in many countries [25,26]. In recent years, their consumption has been steadily increased due to their rich nutritional value and energy-supplying capacity [27]. As a key component of potato tubers, starch accounts for more than 80% of the dry matter [28]. Potato starch synthesis relies on the synergistic interaction of various enzymes and substrates [29]. GBSSI is the most abundant protein on the surface of potato starch granules [30]. Since this enzyme lacks any dedicated CBM, its interaction with glucans involves PTST [17,19]. In this study, we found that StPTST1 directly binds to starch granules (Figure 1). Given that GBSSI interacted with StPTST1 [21], StPTST1 is likely to play a conserved role to mediate the binding of GBSSI to starch granules. Mutation of the *StPTST1* gene resulted in a significant reduction in the transcript abundance of *GBSSI*, accompanied by a concurrent decrease in both amylose content and total starch accumulation in potato tubers. Similarly, disruption of the *MePTST1* gene resulted in a significant reduction in GBSS protein abundance as well as a concurrent decrease in amylose content in cassava plants [31]. In rice, the location of GBSS proteins to starch granules was considerably declined due to the absence of *OsGBP;* in turn, there was less accumulation of amylose and total starch in the *osgbp* mutant [19]. Hence, it is reasonably assumed that StPTST1 is essential for the presence of GBSSI in starch granules to ensure its proper function in amylose biosynthesis. In the chloroplasts of *stptst1* homozygous mutants, the number of starch granules was significantly decreased, and some chloroplasts completely lacked starch granules (Figure S4). This phenotype is consistent with that of *hvptst1* loss-of-function mutants [18]. Surprisingly, the amylose ratio of tuber starch was not dramatically decreased in the *stptst1* mutants. In the *osgbp* mutants, the amylose content was undetectable in the leaves, whereas it was reduced by about 2% in the seeds [19]. This evidence points out the different effects of *ptst1* mutation on starch accumulation in source and sink tissues. On one hand, other proteins that remain unexplored may exert a specific role in influencing GBSS binding to the starch granules in the sink tissues. On the other hand, the mode of starch accumulation is different in source and sink tissues. In leaves, starch is synthesized during the day and is completely degraded at night. In contrast, starch synthesis occurs throughout the enlargement of sink tissues. Therefore, the deficiency of *PTST1* may be partially compensated by the longer duration of amylose accumulation in the sink tissues than that in the source tissues.

Starch chain length characteristics are critical indicators of its molecular structural stability and processing functionality. Pasting properties are shaped by amylose content and amylopectin chain-length distributions [32]. Together, these traits determine starch suitability for diverse applications from food processing to industrial use. In our study, manipulation of *StPTST1* expression profoundly altered these starch properties. Knockout of *StPTST1* reduced tuber amylose content from 17.2% to 15%. It has been documented that low-amylose wheat starch is preferred over regular or amylose-free starch in pasta and noodle production [33,34]. Conversely, overexpression of *StPTST1* impaired the formation of short-chain amylopectin. A reduction in short chains typically enhances starch thermal stability and lowers the risk of granule disintegration during heating, which are advantageous for high-temperature processes like baking and steaming. These changes also decrease amylase hydrolysis efficiency, leading to substantial RS accumulation [35,36,37]. However, the overexpression lines (OE1 and OE4) did not show the expected opposite trend in pasting properties. This likely stems from the mild upregulation of *StPTST1* in tubers upon overexpression; insufficient expression may have prevented detectable shifts in pasting profiles. Beyond amylose content and chain-length distributions, other factors influence pasting properties. For example, granule size impacts wheat starch pasting behavior [38].

In this study, the total starch and amylose contents in the tubers of *stptst1* mutants were significantly reduced (Figure 2), which can meet the food industry’s demand for raw materials with low starch and low amylose contents. In contrast, *StPTST1*-overexpressing plants have greater application potential: their total starch and amylose contents increase simultaneously, and the RS content increases by approximately 3% (Figure 3). As an important prebiotic, RS can alter the composition of gut microbiota and play a key role in preventing gastrointestinal diseases and regulating metabolic disorders caused by fat accumulation [39,40,41]. These results all provide a clear and feasible approach for the targeted improvement in potato quality.

## 4. Materials and Methods

### 4.1. Starch-Binding Assay

*StPTST1* (PGSC0003DMG400030609) was cloned into the pET30a vector to create a fusion protein with a His-tag. The expression of GST-HIS and StPTST1-HIS in BL21 Rosetta cells was induced with 50 mg mL^−1^ of IPTG at 16 °C for 18 h. Fusion proteins were purified using HIS beads, according to the manufacturer’s protocols. For the starch-binding assay, a 200 mg mL^−1^ solution of non-hydrolyzed corn starch was prepared in PBS. The binding of PTST1 fragments (1 μg mL^−1^) and starch (10 mg mL^−1^) was performed at 4 °C by mixing end-over-end for 30 min. Starch was centrifuged for 2 min at 13,000× *g*, and the supernatant was removed. The pellets were washed five times with 500 μL of PBS by gentle vertexing and centrifugation. The starch pellets and supernatants were boiled in an equal volume of SDS-loading buffer before loading.

### 4.2. Engineering of Vectors and Transformation of Potato

The plasmid vector pMDC85:*StPTST1* was generated by In-Fusion^®^HD (Takara Bio Europe SAS, Paris, France). *StPTST1* was cloned into the vector pMDC85, which has a unique *Spe*I restriction site (ACTAGT) and *Kpn*I restriction site (GGTACC) between the promoter and the termination site. *StPTST1* was amplified from Desiree leaf-derived cDNA. The primers used were 5′-CGA CTC TAG AAC TAG TAT GGC TTC ATA CAA TTC AAG AAA AG-3′ as forward and 5′-TCA TTT TTT CTA CCG GTA CCT TCC ACG ACC AAT AAA TTG TTC T-3′ as reverse. The primers used for the cloning procedure were designed following the In-Fusion directives.

For the CRISPR/Cas9, a synthetic guide RNA (sgRNA) (5′-GCA AGC TAA GGA GGA TGC GC-3′) was designed to target the coiled coil domain in StPTST1, while sgRNA (5′-GCT AAA TCT GCT ATC CCA GC-3′) was designed to target before the AMPK1 domain. Two sgRNAs were separately cloned into the entry vector pKSE402, with expression driven by the AtU626 promoter. sgRNA design was performed using the plant gene knockout target design website (https://crispr.dbcls.jp/, accessed on 30 November 2021)

### 4.3. Plant Growth and Molecular Analysis

Binary vectors were transformed into *Agrobacterium tumefaciens* strain GV3101. Desiree (tetraploid potato) and CIP065 (diploid potato) accessions were the respective recipient of the strains harboring the overexpression construct and knockout constructs. Stable transgenic plants were generated via stem segment transformation as described in a reference [42].

Non-transformed Desiree and CIP065 potatoes were used as WT controls. The tubers were collected for starch analyses from transgenic plants and WT after being planted in the greenhouse for 120 days in Kunming, Yunnan, China. The plant aerial portions were allowed to senesce naturally. Five tubers per plant were peeled, chopped into small cubes, and frozen in liquid nitrogen before being stored at −80 °C for total RNA extraction or quantitative real-time PCR (qRT-PCR) assays.

### 4.4. Quantitative Real-Time PCR

A 1 µg aliquot of total RNA was treated with DNase I (Thermo Fisher Scientific Co., Ltd., Shanghai, China) in an 11 µL final volume. Total RNA was extracted using the Plant rapid total RNA Extraction Kit (TIANGEN Biotech Co., Ltd., Beijing, China). EasyScript One-Step gDNA Removal and cDNA Synthesis Supermix (TransGene Biotech Co., Ltd., Beijing, China) was used to synthesize cDNA. qRT-PCR was performed using PerfectStart Green qPCR SuperMix (TransGene Biotech Co., Ltd., Beijing, China). The PCR conditions were as follows: 94 °C for 30 s, followed by 45 cycles of 94 °C for 5 s and 60 °C for 30 s. The *Actin* gene was used as an internal reference gene. The primers are listed in Table S1. Calculations were made according to the equation 2^–∆∆Ct^ [43]. All the analyses were conducted in at least three technical replicates.

### 4.5. Starch Extraction

Freshly cut storage tuber pieces from transgenic and WT plants were suspended in distilled water and homogenized in a 2 L blender. The slurry was filtered through a 100 µm sieve to collect the starch solution. Starch sediments were washed three times with distilled water and dried in a lyophilizer.

### 4.6. Measurement of Total Starch, Amylose, and RS Contents

Total starch and RS contents were measured following AOAC official methods, using the Total Starch (100 A) Kit and Sanshubio determination kit, respectively. Amylose content was determined via the colorimetric method described in reference [44], with a standard curve established using potato amylose standards (Shanghai Yuanye Bio-Technology Co., Ltd., Shanghai, China).

### 4.7. Chain Length Distribution Analysis

Approximately 10 mg of purified starch was resuspended in 5 mL of water containing isoamylase from *Pseudomonas amyloderamosa*. CLD was analyzed via high-performance anion-exchange chromatography with pulsed amperometric detection (HPAEC-PAD), following the protocol provided by Sanshubio Company (Jiangsu Sanshu Biotechnology Co., Ltd., Nantong, Jiangsu, China).

### 4.8. Pasting Property Analysis

For pasting property determination, 1.5 g of pure starch was mixed with 23.5 mL of distilled water in a container. Pasting profiles were measured using a Rapid Visco Analyzer (RVA TecMaster, Newport Scientific, New South Wales, NSW, Australia) following Standard Method 2.

### 4.9. Staining of Starch in Leaves

Leaves from the non-transformed plants and homozygote *stptst1* knockout plants were collected after a 12 h light period. Pigments were removed by extraction in 80% (*v*/*v*) ethanol for 1 h and the leaves were then stained with Lugol’s iodine solution (stock: 250 mg of I_2_, 2.5 g of KI, 125 mL of ddH_2_O, freshly diluted 1000-fold in 100 mM HCl) for 10 min. The Lugol solution was removed after ~5 min and the leaves were washed briefly with 100 mM HCl to remove the excess iodine. White transmission light was used to observe starch granules by microscope.

### 4.10. Statistical Analysis

The data are presented as means ± standard deviation (SD). Results were analyzed using GraphPad Prism 8 software. Results with a corresponding probability value of *p* < 0.05 or *p* < 0.01 were considered statistically significant.

## 5. Conclusions

In this study, the tubers of *StPTST1*-overexpressing plants showed increased amylose and RS contents, which enhances the potential of potato as a nutritionally valuable food for humans. In contrast, the amylose and total starch contents in the tubers of *stptst1* mutants were significantly reduced. The starch fine structure and pasting properties of *StPTST1*-overexpressing plants and *stptst1* mutants were altered to a different degree. Our data indicate that StPTST1 plays a crucial role in mediating the binding of GBSSI to starch granules in leaves and tubers, and influences starch biosynthesis and the nutritional quality of potato.

## Data Availability

In this study, the original contributions presented are included in the main text/Supplementary Materials. For further inquiries, please contact the corresponding author directly.

## Supplementary Materials

The following supporting information can be downloaded at: https://www.mdpi.com/article/10.3390/plants14213351/s1, Figure S1. Ploidy detection of knockout-positive plants. (A) CIP065 serves as the diploid wild-type control. (B) Desiree serves as the tetraploid wild-type control. (C, D) Ploidy level of stptst1 mutants. Theoretically, the DNA content of the tetraploid is twice that of a diploid, which was reflected by the PE-A value of the highest peak on the x-axis. The stptst1 mutants were determined to be diploid. Figure S2. The nucleotide and protein sequences of the edited mutants. (A) The homozygous mutant stptst1-214 has a 4 bp deletion at target site 1 and a 1 bp insertion at target site 2. (B) The heterozygous mutant stptst1-267 has a 3 bp deletion at target site 1 and a 1 bp insertion at target site 2. (C) The amino acid sequence alignment of the stptst1 mutants and WT. The coiled-coil domain (74–118 aa) and CBM48 domain (170–252 aa) are dictated. Figure S3. Tubers from individual potato plants. (A) WT. (B) *stptst1*-214. (C) *stptst1*-267. Scale bar, 3 cm. Figure S4. Analysis of amylose content via staining (A, B) and observation of starch granules in leaves (C, D). (A, C) Control WT leaves. (B, D) *stptst1*-214 knockout leaves. Scale bars, 0.5 cm (A, B) and 50 µm (C, D). Figure S5. Starch physicochemical parameters of *StPTST1*-overexpression lines. No significant differences in PV, TV, FV, BV, and SV were observed between overexpression lines (OE1, OE4) and WT. Data are presented as mean ± SD (n = 3). “ns” indicates no significant difference. Table S1. Primers used in this study.
