# Supplementary material for: Manipulation of StPTST1 Affects Starch Content and Physicochemical Properties of Potato (Solanum tuberosum L.)
Source: Plants (Basel). 2025 Oct 31;14(21):3351. doi: 10.3390/plants14213351 (PMC12610374; doi:10.3390/plants14213351)
Supplement: Supplementary file 1 [file plants-14-03351-s001.zip › plants-3929568-supplementary.pdf]

Supplementary Materials

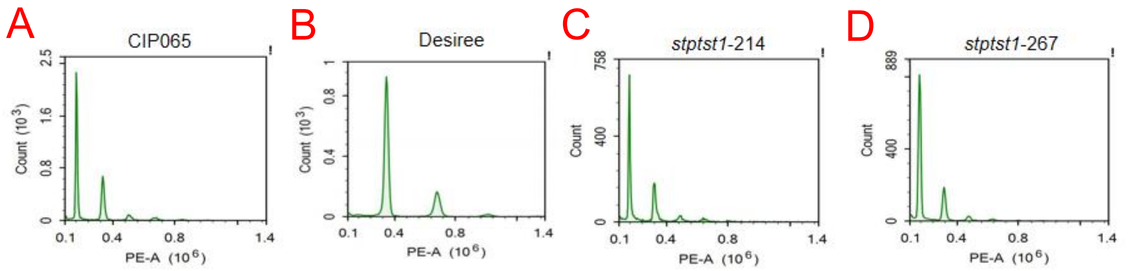

Figure S1. Ploidy detection of knockout positive plants. (A) CIP065 serves as the diploid wild-type control. (B) Desiree serves as the tetraploid wild-type control. (C, D) Ploidy level of *stptst1* mutants. Theoretically, the DNA content of the tetraploid is twice that of a diploid, which was reflected by the PE-A value of the highest peak on the x-axis. The *stptst1* mutants were determined to be diploid.

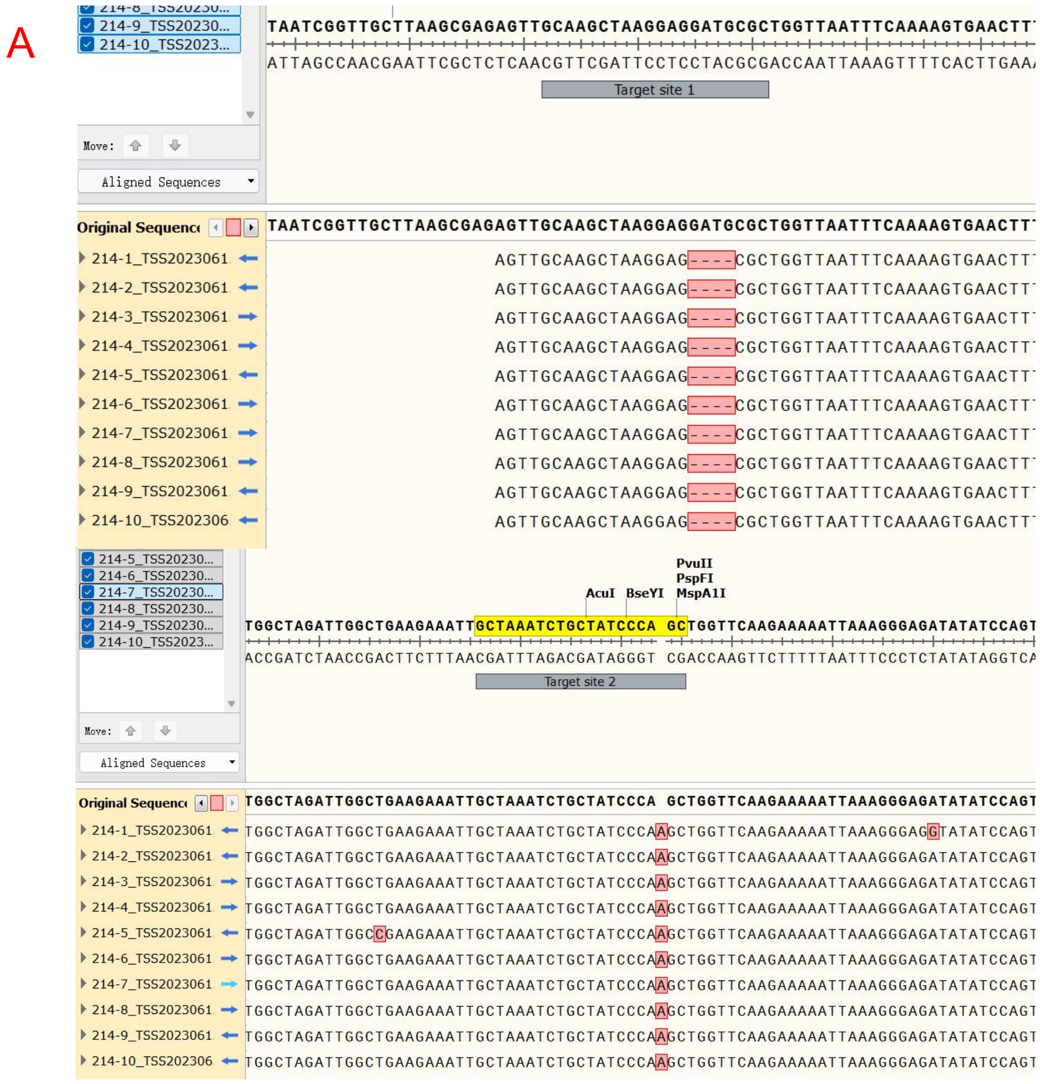

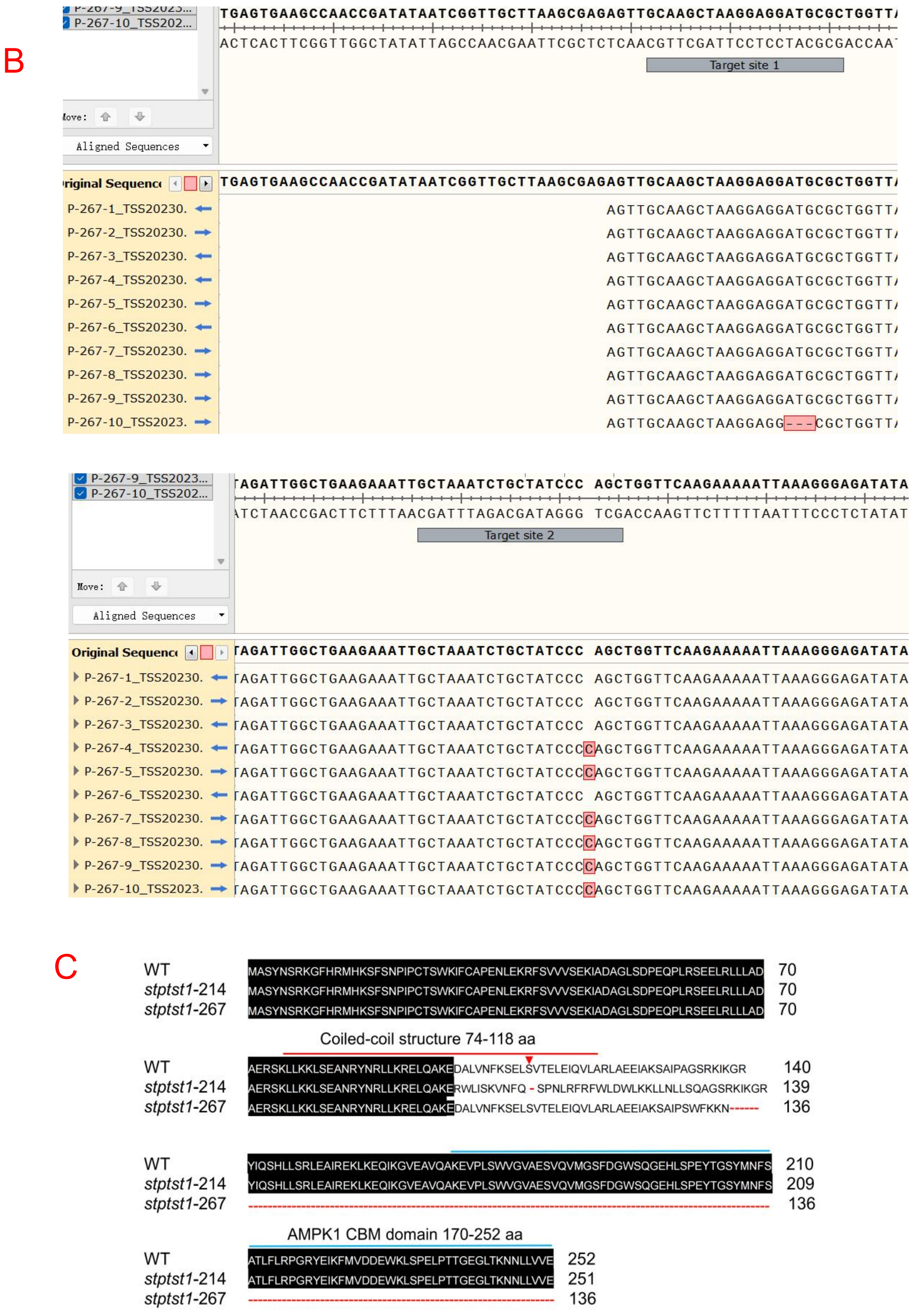

Figure S2. The nucleotide and protein sequences of the edited mutants. (A) The homozygous mutant *stptst1*-214 has a 4-bp deletion at target site 1 and a 1-bp insertion at target site 2. (B)The heterozygous mutant stptst1-267 has a

3-bp deletion at target site 1 and a 1-bp insertion at target site 2. (C) The amino acid sequence alignment of the *stptst1* mutants and WT. The coiled-coil domain (74–118 aa) and CBM48 domain (170–252 aa) are dictated.

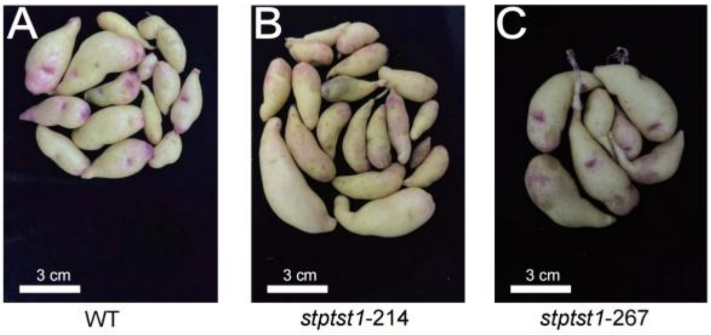

Figure S3. Tubers from individual potato plants. (A) WT. (B) *stptst1*-214. (C) *stptst1*-267. Scale bar, 3 cm.

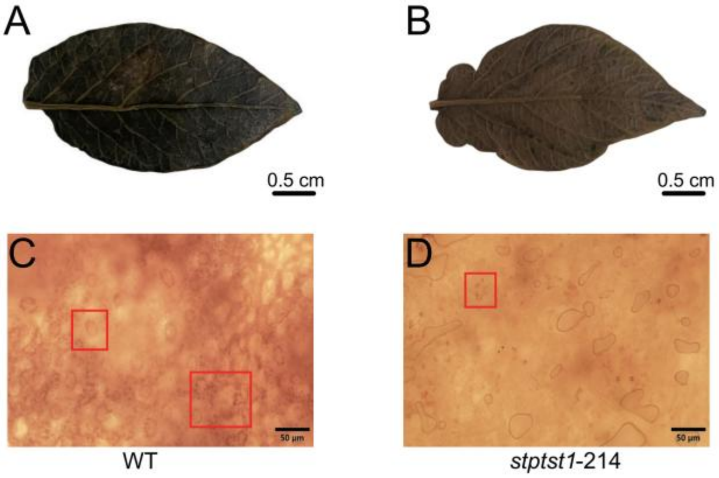

Figure S4. Analysis of amylose content via staining (A, B) and observation of starch granules in leaves (C, D). (A, C) control WT leaves. (B, D) *stptst1*-214 knock-out leaves. Scale bars, 0.5 cm (A, B) and 50 μm (C, D).

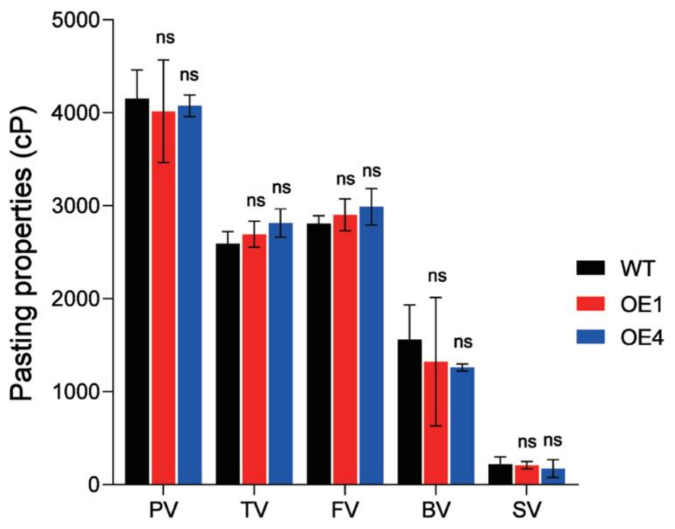

Figure S5. Starch physicochemical parameters of *StPTST1*-overexpression lines. No significant differences in PV, TV, FV, BV and SV were observed between overexpression lines (OE1, OE4) and WT. Data are presented as mean ± SD (n = 3). “ns” indicates no significant difference.
